# Sun Exposure Awareness and Sunscreen Use Among College Students in Saudi Arabia: A Cross-Sectional Analysis

**DOI:** 10.7759/cureus.76146

**Published:** 2024-12-21

**Authors:** Mahdi Al Dhafiri, Nasser A Almulhim, Mudhawi Alsuliman, Saif Y Ashram, Faisal Asiri, Sara Alshanbari, Salma Alshreef, Rahaf Gommosani, Maha Fadel, Hanin A Alfajih

**Affiliations:** 1 Department of Dermatology, King Faisal University, Al-Ahsa, SAU; 2 Department of Medicine and Surgery, King Faisal University, Al-Ahsa, SAU; 3 Department of Medicine, King Faisal University, Al-Ahsa, SAU; 4 Department of Dermatology, King Fahad General Hospital, Jeddah, SAU; 5 Department of Medicine, Prince Sattam bin Abdulaziz University (PSAU), Al-Kharj, SAU; 6 Department of Dermatology, College of Medicine, Umm Al-Qura University, Makkah, SAU; 7 Department of Dermatology, Ibn Sina National College For Medical Studies, Jeddah, SAU; 8 Department of Dermatology, College of Medicine, Qassim University, Qassim, SAU

**Keywords:** awareness, knowledge, practice, skin burns, sun exposure, sunscreen

## Abstract

Background: Excessive sun exposure is a significant risk factor for various skin conditions, including sunburn, premature aging, and skin cancer. This study aimed to assess the awareness, attitudes, and practices regarding sun exposure and sunscreen use among college students in Saudi Arabia.

Methodology: A cross-sectional study was conducted with 388 college students from various disciplines. Data were collected using a structured questionnaire covering demographic information, awareness, and practices related to sun exposure and sunscreen use. Descriptive statistics and chi-square tests were employed to analyze the data.

Results: This study comprised 209 (53.9%) females and 179 (46.1%) males, with the majority aged between 18 and 23 years. Formal education on sun exposure dangers was lacking in 43.0% (167) of participants. The most recognized consequences of excessive sun exposure were sunburn (313, 80.6%) and skin cancer (289, 74.4%). Factors influencing sunscreen use included weather conditions and healthcare provider recommendations. Males were significantly less likely to use sunscreen compared to females. First-year students and participants without formal education on sun exposure were less likely to use sunscreen regularly.

Conclusion: Despite awareness of the risks associated with excessive sun exposure, sunscreen use among college students in Saudi Arabia is inconsistent and influenced by various factors, including gender and education level.

## Introduction

The skin of a human has a surface area of about 1.5-2.0 m^2^, making it the largest organ in the body. It acts as a powerful barrier protecting against harmful effects from foreign substances and environmental pollutants [1]. Sun exposure is a major cause of skin damage. Around half of the Saudi population devotes 10 hours or more to sun exposure each week, exposing themselves to significant ultraviolet (UV) radiation [2]. This excessive sun exposure is linked to various short-term and long-term detrimental effects on the skin [3]. UV exposure is identified as a modifiable risk element for squamous cell carcinoma, basal cell carcinoma, melanoma, and other forms of skin malignancies [3-6]. Over the last 40 years, there has been a consistent rise in the incidence of skin cancer globally, representing one-third of all cancer cases worldwide [7]. Notably, a substantial portion of an individual's lifetime sun exposure occurs during childhood and adolescence [8].

To protect yourself from the sun, avoid exposure between 10:00 am and 2:00 pm, seek shade, and use broad-spectrum sunscreen, along with wide-brimmed hats, protective clothing, and sunglasses [9,10]. Many sun protection programs have been implemented in numerous Western nations to increase public awareness of sun exposure risk and encourage the use of sun protection measures. An increase in awareness among populations has been observed. Nevertheless, compliance with sun protection remains insufficient [11]. In Saudi Arabia, there were few studies on this matter. Not much is known about the awareness of the Saudi population regarding the use of sun protection measures [12]. Information from this study may help design effective interventions.

In 2010, Al Robaee's study in Qassim Province found that although 56% of people were aware of the link between sun exposure and skin cancer, sunscreen use was only 8.3%. Factors associated with sunscreen use included gender, education, and skin type [2]. A 2022 study in Riyadh by Al-Balbeesi et al. showed that while most participants had heard of sunscreen and used it before, awareness gaps existed regarding its proper application and SPF recommendations [10]. More recently, in 2024, Bahashwan's study in the Aseer region revealed that 16.0% of participants used sunscreen regularly, with a total of 74.0% reporting sunscreen use. Increased sun exposure correlated with higher awareness levels of sun damage, influencing sunscreen use [13].

These findings underscore the need for targeted educational efforts to enhance sunscreen awareness and encourage its consistent use among the Saudi population. Raising public awareness is crucial, especially in a country like Saudi Arabia, where the climate predisposes its residents to significant sun exposure.

## Materials and methods

This study was conducted as a descriptive cross-sectional survey utilizing an online-administered questionnaire. This study was conducted between January and June 2024. The cross-sectional design allowed for the collection of data at a single point in time from a specific population. The sample size was calculated using the Raosoft online software (Seattle, WA: Raosoft Inc.), with a 95% confidence level and a margin of error of 5.0%. This resulted in a sample size of 377 participants. To account for potential non-responses, 10% was added, bringing the final sample size to 414. After applying the exclusion criteria, only 388 participants were included in the current study.

All individuals who were older than 18 years, college students residing in Saudi Arabia, and willing to participate were included in the study. Individuals were excluded if they refused to consent, were non-communicative, had intellectual disabilities, or were non-college students younger than 18 years.

A straightforward and clear online questionnaire was distributed among college students in Saudi Arabia. The questionnaire was designed in Arabic, based on a previously validated survey, with items and questions selected from a comprehensive literature review to ensure public comprehension. Participants received an online link that included detailed information about the survey’s objectives, the target population, and a consent form for participation. Upon receiving approval from the institutional research board, the survey was disseminated online through various channels. A sample of the questionnaire was provided in the appendix. A random sampling technique was used, and the questionnaire was distributed online through various social media platforms. This study was conducted among participants in different regions in Saudi Arabia via an online distributed questionnaire.

Data were collected through an online survey designed using Google Forms (Mountain View, CA: Google LLC) and distributed to the target population in Arabic. The questionnaire was self-prepared by the authors depending on the literature review as provided in the appendix. The survey comprised several sections as follows: (1) consent for participation, (2) sociodemographic information, (3) awareness of sun exposure, and (4) sunscreen usage.

The online format facilitated easy access and completion for participants, while the structured sections ensured comprehensive data collection relevant to the study objectives. To validate the self-prepared questionnaire, a pilot study was conducted with a subset of participants from the target population. Based on their feedback, adjustments were made to ensure clarity and accuracy, thus enhancing the validity and reliability of the tool before its full-scale distribution. Acknowledging potential limitations in internet access, we targeted populations with reliable access and promoted the survey through widely accessible online platforms to enhance reach and participation. Participants were reached through targeted online groups, community networks, and educational platforms relevant to the study population, ensuring that respondents met the inclusion criteria for this research.

The data analysis was conducted using SPSS version 26 (Armonk, NY: IBM Corp.) and involved the use of descriptive statistics, chi-square tests, and multivariate logistic regression analysis. Descriptive statistics summarized the demographic characteristics and responses of participants through means, standard deviations, frequencies, and percentages. Chi-square tests were employed to examine the relationships between categorical variables, such as gender, age, educational level, and region, and participants' awareness, attitudes, and practices regarding sun exposure and sunscreen use. Multivariate logistic regression analysis was performed to assess the simultaneous effects of sociodemographic variables, awareness, and attitudes on sunscreen usage. A p-value of less than 0.05 indicated statistical significance.

The ethical approval number (KFU-REC-2024-MAY-ETHICS2411) has been obtained and approved by the Ethical Committee of the Deanship of Scientific Research at King Faisal University. Participation in the study was voluntary. The purpose of the study was stated, and the expected time was reported. Online consent was obtained prior to filling out the survey. To ensure data privacy, all data were stored on the main author’s computer, which was secured with passwords. No participants' private information, such as names or addresses, was collected, and the data were used solely for analysis.

## Results

This study included 388 college students, with a slightly higher representation of females (209, 53.9%). The majority of participants were aged between 18 and 23 years, comprising 68% (264) of the sample. The most prevalent educational levels among participants were the first year (105, 27.1%) and the third and fifth years (both 59, 15.2%). The College of Health Sciences had the highest representation, with 36.3% (141) of participants. Geographically, the participants were most commonly from the Eastern region (100, 25.8%) and the Central region (82, 21.1%) (Table 1).

**Table 1:** Demographic factors of the participants.

Variables		Count	n%
Gender	Male	179	46.1%
	Female	209	53.9%
Age (years)	18-20	132	34.0%
	21-23	132	34.0%
	24-26	111	28.6%
	27-29	9	2.3%
	30 or older	4	1.0%
Educational level	1st year	105	27.1%
	2nd year	42	10.8%
	3rd year	59	15.2%
	4th year	56	14.4%
	5th year	59	15.2%
	6th year	29	7.5%
	Intern	38	9.8%
College	College of Health Sciences (Medicine, Dentistry, Pharmacy, Laboratories, Health Informatics)	141	36.3%
	College of Human Sciences (Arabic/English, History, Geography)	37	9.5%
	College of Science (Mathematics, Physics, Chemistry, Statistics, Biology)	41	10.6%
	Faculty of Sharia (Sharia, Islamic Studies, Regulations and Law)	45	11.6%
	College of Computer Engineering and Science	69	17.8%
	College of Business Administration	51	13.1%
	Other	4	1.0%
Region	Central region	82	21.1%
	Northern region	74	19.1%
	Southern region	63	16.2%
	Eastern region	100	25.8%
	Western region	69	17.8%
Have you received formal education (formal education means: education by schools, universities, or official awareness bodies in the Kingdom of Saudi Arabia) or information about the dangers of exposure to sunlight?	No	167	43.0%
	Yes	158	40.7%
	Not sure	63	16.2%

**Figure 1:**
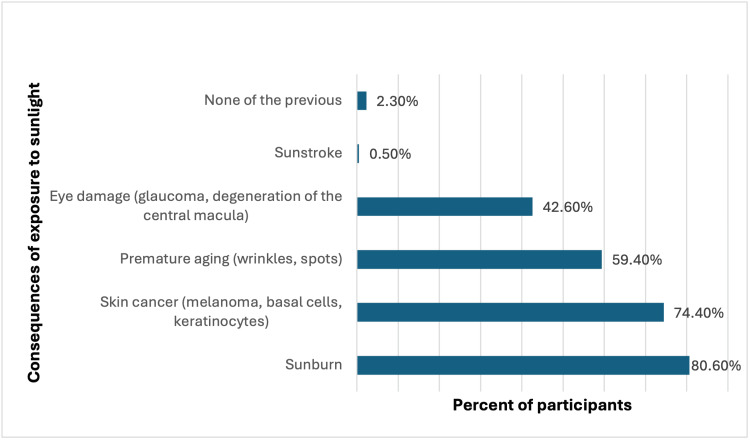
What are the possible consequences of excessive exposure to sunlight? Select all that apply.

The most commonly identified consequences of excessive sunlight exposure were sunburn, cited by 80.6% (313) of participants, and skin cancer (melanoma, basal cells, keratinocytes) mentioned by 74.4% (289). Premature aging (wrinkles, spots) was recognized by 59.4% (230), while 42.6% (165) were aware of the risk of eye damage (glaucoma, degeneration of the central macula) (Figure 1).

Regarding awareness about the effects of varying sun exposure on different skin types and colors, 31.4% (122) of participants reported knowing to some extent, followed by 26.8% with intermediate awareness, and (82) 21.1% with good awareness. When rating their awareness of the harmful effects of skin exposure to sunlight, the most common response was intermediate awareness (158, 40.7%), followed by low awareness (106, 27.8%) and high awareness (73, 18.8%). In terms of sunscreen usage during peak sunlight hours (10 am to 4 pm), 29.9% (116) of participants reported using sunscreen sometimes, 27.3% (106) never used it, and 22.9% (89) used it rarely (Table 2).

**Table 2:** Level of awareness of the participants considering sun exposure and importance of sunscreen.

Awareness assessment		Count	n%
How much do you know about the effects of varying sun exposure on people with different skin types and colors?	I do not know at all	58	14.9%
	I know to some extent	122	31.4%
	Intermediate awareness	104	26.8%
	Good awareness	82	21.1%
	Excellent awareness	22	5.7%
How would you rate your knowledge of the harmful effects of skin exposure to sunlight (e.g., sunburn, skin cancer, premature aging)?	Very low	34	8.8%
	Low	108	27.8%
	Intermediate	158	40.7%
	High	73	18.8%
	Very high	15	3.9%
Do you use sunscreen when you go out during peak sunlight period (10 am to 4 pm)?	Never	106	27.3%
	Rarely	89	22.9%
	Sometime	116	29.9%
	Always	77	19.8%

**Figure 2:**
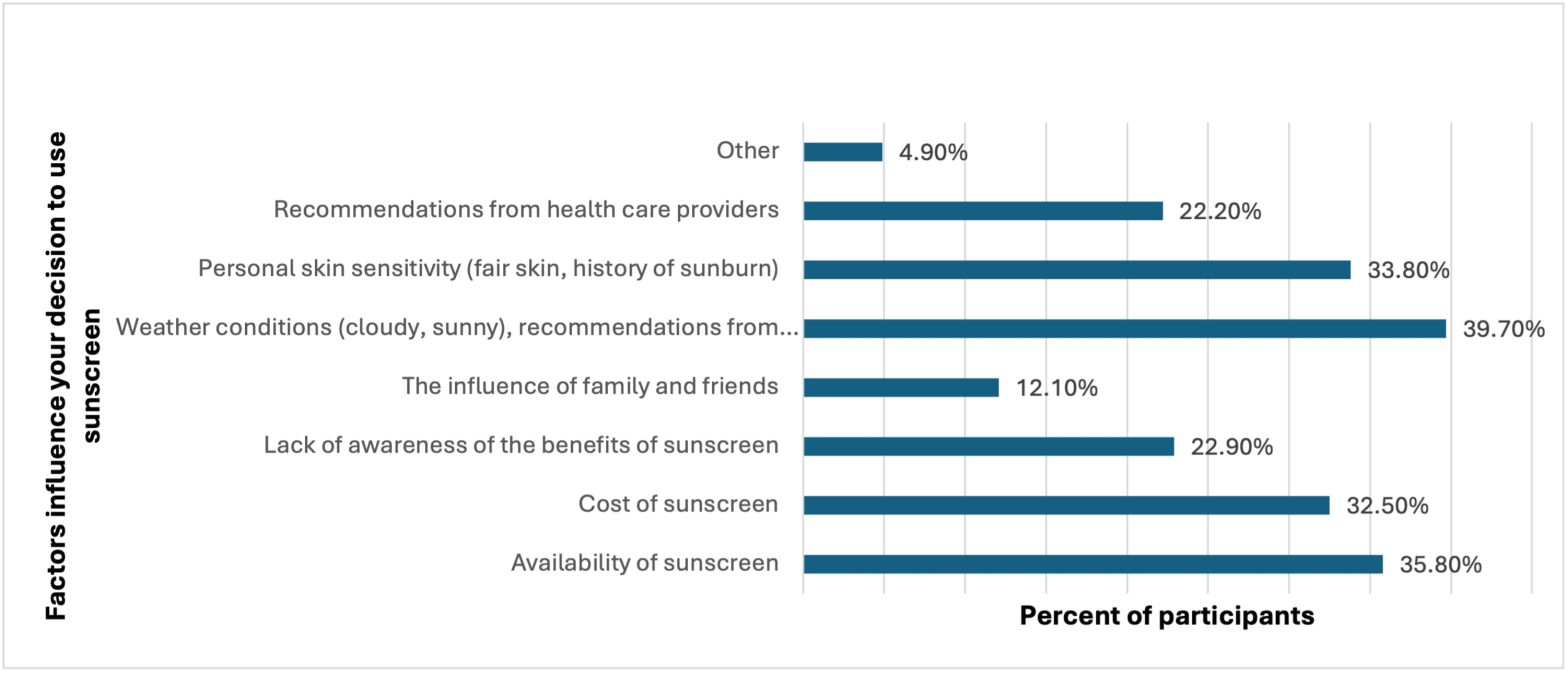
What factors influence your decision to use sunscreen?

Regarding the factors influencing sunscreen use, the most prevalent factors were weather conditions and recommendations from healthcare providers, influencing 39.7% (154) of participants. Other common factors included the availability of sunscreen (139, 35.8%), personal skin sensitivity (131, 33.8%), and the cost of sunscreen (126, 32.5%) (Figure 2).

In terms of sunscreen use practices, 25.8% (100) of participants reported never using sunscreen when outdoors, while 21.9% (85) used it sometimes, and 21.4% (83) used it rarely. Additionally, when asked if wearing a face covering such as a niqab provides adequate sun protection and eliminates the need for sunscreen, 31.4% (122) agreed, while 25.0% (97) remained neutral. Regarding self-examination for unusual skin changes, 57.2% (222) of participants reported never conducting self-examinations. Notably, 78.6% (304) of participants had not been diagnosed with any sun-related skin conditions. Awareness of the sun protection factor (SPF) on sunscreen products was lacking, with 50.8% (197) of participants unaware of its meaning. Conversely, 70.6% (274) believed that regular sunscreen use could reduce the risk of skin cancer. Furthermore, 40.7% (158) considered skin protection from the sun to be extremely important (Table 3).

**Table 3:** Practice considering using of sunscreens.

Sunscreen application and attitude		Count	n%
How often do you use sunscreen when outdoors?	Never	100	25.8%
	Rarely	83	21.4%
	Sometime	85	21.9%
	Most time	75	19.3%
	Every time	45	11.6%
Do you think that wearing a covering over the face such as a niqab provides adequate protection against exposure to the sun's rays and eliminates the need for sunscreen?	Strongly disagree	39	10.1%
	Disagree	66	17.0%
	Neutral	97	25.0%
	Agree	122	31.4%
	Strongly agree	64	16.5%
How often do you self-examine for unusual signs or changes in the skin?	Never	222	57.2%
	Rarely	104	26.8%
	Once a year	15	3.9%
	Every couple of months	32	8.2%
	Every month	15	3.9%
Have you been diagnosed with any skin condition related to sun exposure (such as sunburn, skin cancer)?	No	304	78.6%
	Yes	0	0.0%
	Not sure	83	21.4%
Do you know what the sun protection factor (SPF) on sunscreen products means?	No	197	50.8%
	Yes	85	21.9%
	To some extent, I have a general idea that I am not entirely sure	106	27.3%
Do you think it is important to protect your skin from the sun's rays?	Not completely important	14	3.6%
	Not important	31	8.0%
	Important	81	20.9%
	Very important	104	26.8%
	Extremely very important	158	40.7%
Do you think that using sunscreen regularly can reduce the risk of skin cancer?	No	42	10.8%
	Yes	274	70.6%
	Not sure	72	18.6%

**Figure 3:**
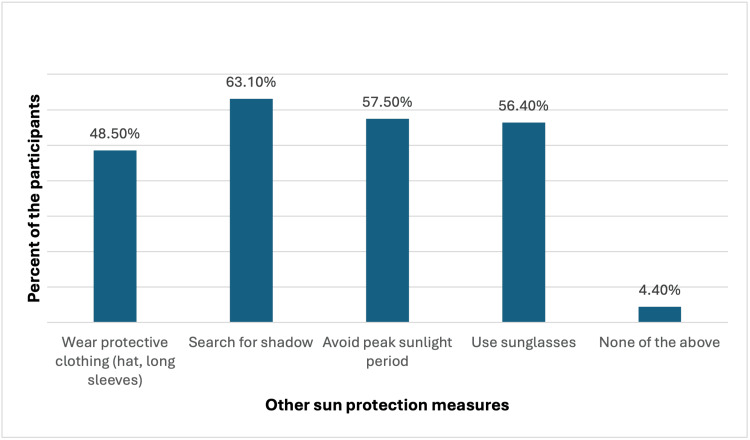
Other than sunscreen, what other sun protection measures do you usually take?

Apart from sunscreen, the most common sun protection measures included seeking shade (245, 63.1%), avoiding peak sunlight periods (223, 57.5%), and wearing sunglasses (219, 56.4%). Protective clothing was also popular, with 48.5% (188) of participants using it (Figure 3).

**Figure 4:**
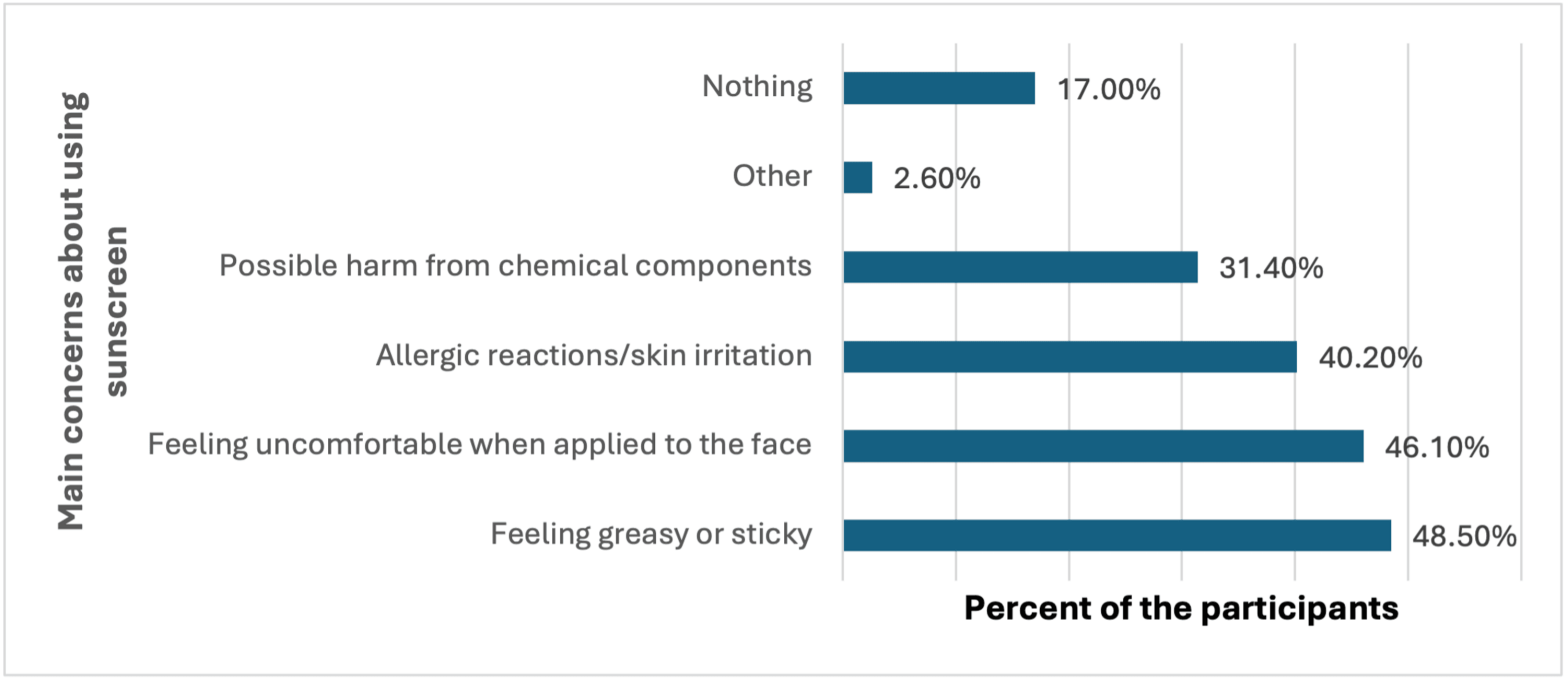
What are your main concerns about using sunscreen?

Concerns about using sunscreen were prevalent, with 48.5% (188) of participants feeling it was greasy or sticky, 46.1% (179) feeling uncomfortable when applied to the face, and 40.2% (156) worried about allergic reactions or skin irritation (Figure 4).

This study found significant gender differences in sunscreen use, with females more likely than males to apply sunscreen consistently. Age also played a role, with younger age groups (18-23 years) showing moderate use, whereas those 30 years and older were the least likely to use sunscreen. Educational level influenced usage patterns, with first-year students showing the highest rates of non-use, while those in later years or with formal education on sun exposure risks were more consistent in their sunscreen use (Table 4).

**Table 4:** The relation between sunscreen usage and demographic factors. *Significant p-values.

Demographic factors		How often do you use sunscreen when outdoors?										
		Never		Rarely		Sometime		Most time		Every time		p-Value
		Count	n%	Count	n%	Count	n%	Count	n%	Count	n%	
Gender	Male	80	44.7%	41	22.9%	23	12.8%	27	15.1%	8	4.5%	0.000*
	Female	20	9.6%	42	20.1%	62	29.7%	48	23.0%	37	17.7%	
Age (years)	18-20	47	35.6%	23	17.4%	21	15.9%	24	18.2%	17	12.9%	0.035*
	21-23	23	17.4%	32	24.2%	39	29.5%	19	14.4%	19	14.4%	
	24-26	25	22.5%	27	24.3%	23	20.7%	28	25.2%	8	7.2%	
	27-29	3	33.3%	1	11.1%	1	11.1%	3	33.3%	1	11.1%	
	30 or older	2	50.0%	0	0.0%	1	25.0%	1	25.0%	0	0.0%	
Educational level	1st year	46	43.8%	16	15.2%	16	15.2%	16	15.2%	11	10.5%	0.000*
	2nd year	8	19.0%	8	19.0%	15	35.7%	4	9.5%	7	16.7%	
	3rd year	8	13.6%	17	28.8%	13	22.0%	13	22.0%	8	13.6%	
	4th year	4	7.1%	16	28.6%	15	26.8%	12	21.4%	9	16.1%	
	5th year	15	25.4%	11	18.6%	16	27.1%	16	27.1%	1	1.7%	
	6th year	6	20.7%	7	24.1%	6	20.7%	5	17.2%	5	17.2%	
	Intern	13	34.2%	8	21.1%	4	10.5%	9	23.7%	4	10.5%	
College	College of Health Sciences (Medicine, Dentistry, Pharmacy, Laboratories, Health Informatics)	34	24.1%	34	24.1%	29	20.6%	28	19.9%	16	11.3%	0.156
	College of Human Sciences (Arabic/English, History, Geography)	5	13.5%	6	16.2%	14	37.8%	8	21.6%	4	10.8%	
	College of Science (Mathematics, Physics, Chemistry, Statistics, Biology)	11	26.8%	8	19.5%	4	9.8%	10	24.4%	8	19.5%	
	Faculty of Sharia (Sharia, Islamic Studies, Regulations and Law)	21	46.7%	7	15.6%	8	17.8%	6	13.3%	3	6.7%	
	College of Computer Engineering and Science	19	27.5%	17	24.6%	16	23.2%	13	18.8%	4	5.8%	
	College of Business Administration	10	19.6%	10	19.6%	13	25.5%	9	17.6%	9	17.6%	
	Other	0	0.0%	1	25.0%	1	25.0%	1	25.0%	1	25.0%	
Region	Central region	13	15.9%	15	18.3%	21	25.6%	20	24.4%	13	15.9%	0.037
	Northern region	11	14.9%	18	24.3%	18	24.3%	15	20.3%	12	16.2%	
	Southern region	14	22.2%	17	27.0%	14	22.2%	12	19.0%	6	9.5%	
	Eastern region	39	39.0%	18	18.0%	22	22.0%	15	15.0%	6	6.0%	
	Western region	23	33.3%	15	21.7%	10	14.5%	13	18.8%	8	11.6%	
Have you received formal education or information about the dangers of exposure to sunlight?	No	47	28.1%	31	18.6%	38	22.8%	39	23.4%	12	7.2%	0.067
	Yes	34	21.5%	36	22.8%	35	22.2%	25	15.8%	28	17.7%	
	Not sure	19	30.2%	16	25.4%	12	19.0%	11	17.5%	5	7.9%	

The regression analysis presented in Table 5 reveals significant associations between specific demographic factors and the likelihood of using sunscreen regularly. Female respondents were significantly more likely to use sunscreen than males (OR = 18.0, 95% CI = 7.46-45.86, p < 0.001). Among age groups, only the 21-23 years group showed a statistically significant increase in sunscreen use compared to the 18-20 years reference group (OR = 2.28, 95% CI = 1.00-5.20, p = 0.049). Educational level showed a strong positive trend, with increased sunscreen use seen from the second to fourth years, with the fourth year showing the highest likelihood (OR = 9.04, 95% CI = 2.44-36.26, p = 0.001). Geographically, respondents from the Eastern region were significantly less likely to use sunscreen compared to those from the Central region (OR = 0.15, 95% CI = 0.04-0.48, p = 0.001) (Table 5).

**Table 5:** Regression analysis of factors affecting using of sunscreen comparing always to never use. *Significant p-values.

Variables		Odd ratio 95% confidence interval	p-Value
Gender	Male	Reference	
	Female	18.0 (7.46-45.860)	0.001*
Age (years)	18-20	Reference	
	21-23	2.28 (1.00-5.199)	0.049*
	24-26	0.88 (0.33-2.33)	0.804
	27-29	0.92 (0.08-9.47)	0.945
	30 or older	0.54 (0.02-11.87)	0.698
Educational level	1st year	Reference	
	2nd year	3.65 (1.09-12.26)	0.035*
	3rd year	4.18 (1.28-13.61)	0.017*
	4th year	9.04 (2.44-36.26)	0.001*
	5th year	0.28 (0.03-2.34)	0.239
	6th year	3.48 (0.89-13.53)	0.071
	Intern	1.28 (0.35-4.71)	0.7038
Region	Central region	Reference	
	Northern region	1.09 (0.35-3.35)	0.879
	Southern region	0.42 (0.13-1.46)	0.176
	Eastern region	0.15 (0.04-0.48)	0.001*
	Western region	0.34 (0.11-1.05)	0.063

## Discussion

The current study provides valuable insights into sunscreen usage behaviors and the level of awareness regarding the risks of sun exposure among college students. These findings are critical as they highlight gaps in awareness and practice, informing future educational interventions aimed at reducing the incidence of sun-related health issues, particularly skin cancer.

Gender differences in sunscreen use

The data reveals significant gender differences in sunscreen use. Specifically, females reported a higher prevalence of sunscreen use than males similar to what was reported by previous research indicating that females are generally more aware of and proactive about skin protection measures compared to males [14-17]. The higher usage of sunscreen among females could be attributed to greater awareness and societal norms that emphasize skin care for women [2,13].

Age-related variations in sunscreen use

Participants aged 18-20 years and 21-23 years were more likely to use sunscreen sometimes or most of the time. This trend could be explained by increased health awareness and access to information among younger students, who are often more exposed to educational campaigns and social media messages regarding sun safety [13,14]. In contrast, participants aged 30 years or older were the least likely to use sunscreen regularly, with 50.0% (2) never using it. This lower rate of usage among older participants may be due to a lack of targeted educational efforts towards this age group or ingrained habits formed over the years [18,19].

Impact of formal education on sunscreen use

Formal education about the dangers of sun exposure appears to positively influence sunscreen use. This underscores the importance of formal educational programs in promoting healthy behaviors [20,21]. However, the non-significance level indicates that while there is a positive trend, it is not strong enough to conclusively affirm the impact without further investigation. Enhancing the quality and reach of educational interventions could potentially improve these outcomes.

Awareness of sun exposure risks

This study also assessed participants' awareness about the effects of sun exposure and their awareness of the importance of sunscreen. While almost one-third of participants reported knowing to some extent about the effects of varying sun exposure on different skin types and colors, only 5.7% (22) claimed excellent awareness. Additionally, 40.7% (158) rated their awareness of the harmful effects of skin exposure to sunlight as intermediate, indicating a moderate level of awareness. This gap in awareness suggests that there is room for improvement in educational efforts to enhance understanding of sun-related risks and the importance of preventive measures like sunscreen use.

Barriers to sunscreen use

This study identified several barriers to regular sunscreen use, including the perception that it is greasy or sticky, discomfort when applied to the face, and concerns about allergic reactions or skin irritation. These concerns are consistent with previous research highlighting common deterrents to sunscreen use [2,17,22,23]. Addressing these barriers through the development of more user-friendly sunscreen formulations and better education about the importance of sunscreen can help increase its usage. For example, promoting sunscreens that are non-greasy, hypoallergenic, and suitable for sensitive skin could alleviate some of these concerns.

Importance of sun protection measures

Apart from sunscreen, other sun protection measures such as seeking shade, avoiding peak sunlight periods, and wearing sunglasses were also commonly practiced by participants. This holistic approach to sun protection is crucial as it reduces overall exposure to harmful UV rays [24]. Encouraging a combination of protective behaviors, rather than relying solely on sunscreen, can enhance the effectiveness of sun safety strategies [25,26].

The findings of this study hold significant implications for both clinical practice and public health initiatives aimed at reducing the burden of sun-related health issues, including skin cancer. From a clinical perspective, these insights can inform improved prevention strategies by enabling dermatologists and primary healthcare providers to target at-risk groups, such as males and older adults, with tailored educational campaigns. Personalized counseling on sun protection can address specific barriers such as concerns over sunscreen greasiness or skin irritation, with recommendations for hypoallergenic or non-comedogenic formulations. Additionally, routine health consultations can incorporate sun safety advice, particularly in regions with high UV exposure. Public health efforts can benefit from targeted awareness campaigns that address gender- and age-specific disparities in sunscreen use. Policies that promote accessible and affordable sunscreen, potentially subsidized in high-risk areas, may enhance compliance. Moreover, integrating sun safety education into school and university curriculums can encourage early adoption of protective behaviors. Beyond sunscreen use, public health messaging should emphasize comprehensive sun protection measures, including wearing protective clothing and sunglasses and seeking shade during peak sunlight hours.

This study has several limitations. One major limitation is its reliance on self-reported data, which is subject to recall bias and social desirability bias, potentially leading to inaccuracies in reporting sunscreen use and sun protection behaviors. This study population is limited to college students, which may not fully represent the general population or other age groups, thereby affecting the generalizability of the findings. Furthermore, the cross-sectional design of this study prevents establishing causal relationships between factors such as education, awareness, and sunscreen use. The scope of this study is also limited as it does not extensively explore other potential determinants, such as socioeconomic status, skin type, or geographic UV exposure levels. Finally, the geographical focus on a specific region may limit the applicability of the findings to populations in different climatic or cultural settings.

Despite these limitations, this study has notable strengths. The large sample size enhances the statistical power and reliability of the findings. The study’s comprehensive approach, assessing factors such as gender, age, educational background, and barriers to sunscreen use, provides a holistic understanding of the issue. Its focus on a population with high exposure to educational campaigns and social media offers insights relevant to designing interventions targeting younger, digitally connected audiences. Additionally, by identifying practical barriers to sunscreen use, such as discomfort or concerns about skin irritation, this study provides actionable recommendations for product developers and healthcare educators. Finally, its emphasis on behavioral trends, rather than merely assessing awareness, delivers a more accurate picture of adherence to sun protection recommendations, making the findings valuable for both clinical and public health applications.

## Conclusions

In conclusion, this study highlights significant gender, age, educational level, and geographical differences in sunscreen use among college students. While females, younger participants, and those with formal education about sun exposure are more likely to use sunscreen regularly, significant gaps remain, particularly among males and older students. Addressing these disparities through targeted educational interventions and addressing barriers to sunscreen use can improve sun protection behaviors. Future research should explore the effectiveness of different educational strategies and the development of user-friendly sunscreen products to enhance compliance and reduce the risk of sun-related health issues.

## Appendices

The questionnaire

A. Gender

1. Male

2. Female

B. Age (years)

1. 18-20

2. 21-23

3. 24-26

4. 27-29

5. 30 or older

C. Educational level

1. 1st year

2. 2nd year

3. 3rd year

4. 4th year

5. 5th year

6. 6th year

7. Intern

D. College

1. College of Health Sciences (Medicine, Dentistry, Pharmacy, Laboratories, Health Informatics)

2. College of Human Sciences (Arabic/English, History, Geography)

3. College of Science (Mathematics, Physics, Chemistry, Statistics, Biology)

4. Faculty of Sharia (Sharia, Islamic Studies, Regulations and Law)

5. College of Computer Engineering and Science

6. College of Business Administration

7. Other

E. Region

1. Central region

2. Northern region

4. Southern region

5. Eastern region

6. Western region

F. Have you received formal education (formal education means: education by schools, universities, or official awareness bodies in the Kingdom of Saudi Arabia) or information about the dangers of exposure to sunlight?

1. No

2. Yes

3. Not sure

G. What are the possible consequences of excessive exposure to sunlight? You could choose more than one answer.

1. Sunburn

2. Skin cancer (melanoma, basal cells, keratinocytes)

3. Premature aging (wrinkles, spots)

4. Eye damage (glaucoma, degeneration of the central macula)

5. Sunstroke

6. None of the previous

H. How much do you know about the effects of varying sun exposure on people with different skin types and colors?

1. I do not know at all

2. I know to some extent

3. Intermediate knowledge

4. Good knowledge

5. Excellent knowledge

I. How would you rate your knowledge of the harmful effects of skin exposure to sunlight (e.g., sunburn, skin cancer, premature aging)?

1. Very low

2. Low

3. Intermediate

4. High

5. Very high

J. Do you use sunscreen when you go out during peak sunlight period (10 am to 4 pm)?

1. Never

2. Rarely

3. Sometimes

4. Always

K. What factors influence your decision to use sunscreen? You can choose more than one answer.

1. Availability of sunscreen

2. Cost of sunscreen

3. Lack of awareness of the benefits of sunscreen

4. The influence of family and friends

5. Weather conditions (cloudy, sunny)

6. Personal skin sensitivity (fair skin, history of sunburn)

7. Recommendations from healthcare providers

8. Other

L. How often do you use sunscreen when outdoors?

1. Never

2. Rarely

3. Sometimes

4. Most time

5. Every time

M. Do you think that wearing a covering over the face such as a niqab provides adequate protection against exposure to the sun's rays and eliminates the need for sunscreen?

1. Strongly disagree

2. Disagree

3. Neutral

4. Agree

5. Strongly agree

N. How often do you self-examine for unusual signs or changes in the skin?

1. Never

2. Rarely

3. Once a year

4. Every couple of months

5. Every month

O. Have you been diagnosed with any skin condition related to sun exposure (such as sunburn or skin cancer)?

1. No

2. Yes

3. To some extent, I have a general idea that I am not entirely sure

P. Do you think it is important to protect your skin from the sun's rays?

1. Not completely important

2. Not important

3. Important

4. Very important

5. Extremely very important

Q. Do you think that using sunscreen regularly can reduce the risk of skin cancer?

1. No

2. Yes

3. Not sure

R. Other than sunscreen, what other sun protection measures do you usually take? You can choose more than one answer.

1. Wear protective clothing (hat, long sleeves)

2. Search for shadow

3. Avoid peak sunlight period

4. Use sunglasses

5. None of the above

S. What are your main concerns about using sunscreen? You can choose more than one answer.

1. Feeling greasy or sticky

2. Feeling uncomfortable when applied to the face

3. Allergic reactions/skin irritation

4. Possible harm from chemical components

5. Other

6. Nothing
